# Synopsis of a Report on Osteoporosis1Read before the annual meeting of the Pennsylvania State Veterinary Medical Association, March, 1899.

**Published:** 1899-06

**Authors:** C. J. Marshall

**Affiliations:** Philadelphia, Pa.


### SYNOPSIS OF A REPORT ON OSTEOPOROSIS.[1]

By C. J. Marshall, V.M.D.,
PHILADELPHIA, PA.

Last year a set of questions on the subject of osteoporosis was sent to the veterinarians of this State in order to ascertain some facts in reference to the disease. The questions—ten in number —were as follows :

1. Does osteoporosis exist in your locality ?
2. If so, how many cases can you recall, and how many died or were destroyed on account of this disease ?

[1] Read before the annual meeting of the Pennsylvania State Veterinary Medical Association, March, 1899.

3. How many cases terminated in recovery? Complete or partial? How were they treated?

4. Do you advise treatment, and what?

5. Do you know any causes for this disease?

6. Do you consider it contagious?

7. In examination for soundness, what symptoms do you consider sufficient to condemn the animal?

8. Were the cases that you observed in towns or on farms?

9. Did the disease develop in old or young animals?

10. Were the subjects fat or thin, and what sex?

Answers to these questions were received from sixty-five veterinarians, representing nearly all portions of the State. These reports show that the disease is comparatively rare in all sections of the State except Philadelphia and the immediate vicinity. Quite a number of cases were reported from near Pittsburg. With these two localities excepted, there were only 127 cases reported. This covers all the cases observed by these men since they have been in practice. Only six cases were reported as having died from this disease. Very few cases were completely cured, while quite a number of partial cures are reported. The jaws remained large, but the animals were able to work. Nearly all advise treatment in mild cases where the animal is valuable. Nearly all advise green food, pasture if possible, in a locality where the disease is rare. In times where green foods are not attainable nearly all recommend medical treatment—calcium phosphate, iron, and iodide of potash were especially mentioned; also counter-irritation where lameness existed.

Question 5, in reference to the cause of the disease, was answered no by nearly all. One veterinarian thinks it to be identical to actinomycosis and due to the same cause. By one observer the two diseases have existed badly at the same time on the same farm. One of our good men thinks we have no such disease; that it is only a symptom of other diseases, as osteomyelitis, actinomycosis, tuberculosis, glanders, etc.

By none is it considered contagious. Any well-marked symptom of the disease is conceded by the majority to be sufficient to condemn the animal in an examination for soundness. The majority of cases observed were in towns or in cities. Age made but little difference, and ranged from four weeks to twenty years. The animals were mostly in good condition and well fed. As to sex, they were about equally divided.

The following reported cases with histories:

| | Quest. 1 | Quest. 2 | Quest. 3 | Quest. 4 | Quest. 5 | Quest. 6 | Quest. 7 | Quest. 8 | Quest. 9 | Quest. 10 |
|---|---|---|---|---|---|---|---|---|---|---|
| Dr. Charles Leinhart, Wayne. | Yes | One, destroyed. | None; iron quinine. | None. | No | No | Enlarged jaw, emaciation, lame. | Small town. | 5 years old. | Good condition; male. |
| W. H. Ridge, Trevose, Bucks Co. | No | One, living. | One, partial; pasture. | Pasture in section free from the disease. | No | ? | Thickening of jaws. | From the city. | Old animals. | |
| Ira M. Gregg, Garwood. | Yes | Seven. | Two, partial; pasture. | Pasture; hard water. | No | No | .............. | On farms. | Young. | |
| Wm. P. Phipps, Lionville, Chester Co. | Yes | One, destroyed. | None. | ............ ..... | No | No | Enlarged jaw and lameness. | In country. | 9 years old. | Poor; male. |
| F. F. Hoffman, Brookville. | Yes | Three; one. | Two, partial. | One not treat'd; one sent to pasture. | No | No | Any symptom. | | | |
| J. R. Keelor, Harleysville. | Yes | One, one. | .................. | Change food, and give tonics. | Food deficient in lime-salts. | No | Any symptom. | One; farm. | Young. | Good; male. |
| H. J. S. Weicksel, Shamokin. | Yes | Three; one. | None. | In bad cases no treatment. | No | ? | Any symptom. | One, farm; two city. | Aged. | Fair; two geldings, one mare. |
| W. B. Collom, Doylestown. | Yes | One. | .................. | Calcium phosphate; counter-irritation. | No | No | | | | |
| J. A. Haas, Girard | Yes | One, destroyed. | None. | .................. | No | Yes | Enlargement of the jaws. | In town. | Middle age. | Thin; female. |
| W. H. Mattson, Camp Ground. | Yes | Twelve, one killed. | Eight; one partial. | Wheat-bran, pasture, and iod. of potash. | Abstinence from green food. | ? | Enlargement of the jaws. | .............. | Eight to ten. | Fair. |
| John L Bradley, Mercersburg. | Yes | One. | Partial; iod. of potash. | Iod. of potash, bran mashes, and iron. | No | No | .............. | In country. | Young. | Fair; mare. |
| B. M. Underhill, Media. | Yes | Ten. | None. | Oats, clover, hay, pasture, bone-meal, arsenic. | No | No | Any symptom. | On farms. | Young. | Good. |
| Lewis A. Kline, Lewistown. | Yes | Two. | .................. | Iod. of potash. | No | ? | .............. | In town. | Old. | |
| J. R. Phillips, North East. | Yes | Consider it the same as actinomycosis in cattle. | | | | | | | | |
| S. J. J. Harger, Philadelphia. | Yes | Many. | One partial, none absolute. | Pasture, bone-meal, lime-water. | No | No | Any symptom. | Both. | All ages. | Equal. |

| | Quest. 1 | Quest. 2 | Quest. 3 | Quest. 4 | Quest. 5 | Quest. 6 | Quest. 7 | Quest. 8 | Quest. 9 | Quest. 10 |
|---|---|---|---|---|---|---|---|---|---|---|
| James A. Waugh, Pittsburg. | Yes | Many; one died, one destroyed. | ................ | Iron, potash, salts, and pasture. | No | No | Enlargement of the jaws, lameness. | Both. | All ages. | Both; Both. |
| A. W. Hinman, Braddock. | Yes | Three died; one destroyed; one died from other causes. | One, partial. | Iron and pasture; counter-irritation. | No | No | Any symptom. | Town. | Young and old. | Both. |
| Warren T. Edwards, Downingtown. | Yes | One, destroyed. | ....... ........ | Iod. of potash | No | ? | Any symptom. | Town. | Middle age; fat. | |
| J. H. Oyler, Harrisburg. | Yes | Two, destroyed. | ................ | Iod. of potash. | No | No | Any symptom. | City. | 9 years and 8 years. | Fat; geldings. |
| Charles Goentner, Bryn Mawr. | Yes | 200; about five or six. | Nearly 95 p. ct. partially, 60 per ct. completely. | Yes. | No | ? | Any symptom. | Both. | From 4 wks. to 20 years. | Both. |
| 27 C. Lintz, Philadelphia. | Yes | Three. | One died, one destroyed, other sold. | Grass in season, only hay out of season. | Too much grain, no exercise. | No | Lameness only. | Both. | 7 to 20. | Both. |
| A. F. Schreiber, Philadelphia. | Yes | Many; five deaths. | Ten, partial; grass. | Iod. of potash, or grass. | No | Bad where actinomycosis is bad. | No | Any symptom. | Both. | Both. |
| E. Mayhew Michener, North Wales. | Yes | 33; eleven di'd or were destroyed; thirteen passed out of view. | Nine, partial. | Green food if possible, counter-irritation if lame; disinfect stable; but little grain. | Yes | No | No | Any symptom. | Both. | Both. |
| M. E. Conard, Westgrove. | Yes | Three; one destroyed. | Could not be traced. | Pasture. | No | No | Thickened jaw. | Both. | Middle age. | Good condition. |
| B. H. Detwiler, per W. F. Tomlinson, Williamsport. | Yes | Four, three killed. | One, partial. | Yes, in. | No | No | Enlarged jaw, peculiar stiffness. | Town. | Both. | Both. |
| Dr. M Moriarty, Gettysburg. | Yes | Five, two partial. | | ................ | Thinks it is hereditary. | .............. | .............. | Town. | Both. | Both. |

The following wrote that osteoporosis did not exist in their neighborhood:

Dr. B. F. Minich, Columbia (none in twelve years' practice); W. M. Briggs, Gettysburg; H. D. Entrekin, Kennett Square (case brought from city seemed to improve); R. P. Feiser, East Berlin; J. F. Butterfield, Montrose; W. C. Lazarus, Stroudsburg; Harry Hayward, State College; S. P. Bishop, Carlisle; Harry McNeal, Milton; A. H. Balliet, Ballietsville; D. E. Kimmell, Brownville; Elmer E. Seitz, Seitzville, York County; George K. Swank, Mauch Chunk; Elias Snyder, Orwigsburg; John Bennett, Ambler; H. K. Walter, Wilkesbarre; E. E. Bittles, New Castle, Lawrence County; Jesse Z. Hillegass, Red Hill; J. W. Reed, Danville; E. E. Porter, New Castle; Edwin Hogg, Kirkwood, Lancaster County; N. E. Reinhart, Pottstown (has seen three cases in other places); H. P. Bock, Phillipsburg; J. C. Kingsland, Canton, Bradford County; F. J. McNeal, Hazelton; S. B. Willard, Yardley; J. G. Bethune, Punxsutawney; J. B. Irons, Erie; Otto Noack, Reading (thinks that there is no such disease—only found in osteomyelitis, actinomycosis, etc.).

Eight reports were not signed and contained no new information.

